# Cardioprotective effects of pharmacological blockade of the mitochondrial calcium uniporter on myocardial ischemia-reperfusion injury^1^

**DOI:** 10.1590/s0102-865020200030000006

**Published:** 2020-05-20

**Authors:** Francisco Sandro Menezes-Rodrigues, José Gustavo Padrão Tavares, Enio Rodrigues Vasques, Paolo Ruggero Errante, Erisvaldo Amarante de Araújo, Marcelo Pires-Oliveira, Carla Alessandra Scorza, Fúlvio Alexandre Scorza, Murched Omar Taha, Afonso Caricati-Neto

**Affiliations:** I Fellow PhD degree, Postgraduate Program in Pharmacology, Universidade Federal de São Paulo (UNIFESP), Brazil. Conception and design of the study, analysis and interpretation of data, manuscript writing.; II Fellow PhD degree, Laboratory of Liver Surgery and Transplant (LIM/37), School of Medicine, Universidade de São Paulo (USP), Brazil. Analysis and interpretation of data.; III Fellow PhD degree, Postgraduate Program in Pharmacology, UNIFESP, Sao Paulo-SP, Brazil. Analysis and interpretation of data, manuscript writing.; IV Fellow MSc degree, Postgraduate Program in Pharmacology, UNIFESP, Sao Paulo-SP, Brazil. Analysis and interpretation of data.; V PhD, Assistant Professor, School of Medicine, União Metropolitana de Educação e Cultura (UNIME), Lauro de Freitas-BA, Brazil. Manuscript preparation and writing.; VI PhD, Associate Professor, Department of Neurology and Neurosurgery, UNIFESP, Sao Paulo-SP, Brazil. Critical revision.; VII PhD, Associate Professor, Department of Surgery, UNIFESP, Sao Paulo-SP, Brazil. Technical procedures, interpretation of data.; VIII PhD, Associate Professor, Department of Pharmacology, UNIFESP, Sao Paulo-SP, Brazil. Conception and design of the study, critical revision, final approval.

**Keywords:** Myocardial Reperfusion Injury, Ischemia, Calcium, Rats

## Abstract

**Purpose:**

To evaluate whether the attenuation of mitochondrial Ca^2+^ overload produced by pharmacological blockade of mitochondrial Ca^2+^ uniporter (MCU) protects the myocardium against injuries caused by cardiac ischemia and reperfusion (CIR).

**Methods:**

CIR was induced in adult male Wistar rats (300-350 g) by occlusion of the left anterior descendent coronary artery (10 min), followed by reperfusion (120 min). Rats were treated with different doses of MCU blocker ruthenium red (RuR), administered 5 min before ischemia or reperfusion.

**Results:**

In untreated rats, the incidences of ventricular arrhythmias (VA), atrioventricular block (AVB) and the lethality (LET) induced by CIR were 85%, 79% and 70%, respectively. In rats treated with RuR before ischemia, the incidences of VA, AVB and LET were significantly reduced to 62%, 25% and 25%, respectively. In rats treated with RuR after ischemia, the incidences of VA, AVB and LET were significantly reduced to 50%, 25% and 25%, respectively.

**Conclusion:**

The significant reduction of the incidence of CIR-induced VA, AVB and LET produced by the treatment with RuR indicates that the attenuation of mitochondrial Ca^2+^ overload produced by pharmacological blockade of MCU can protect the myocardium against injuries caused by CIR.

## Introduction

Acute myocardial infarction (AMI), which is caused by cardiac ischemia, has become one of the leading causes of death in Western countries and is becoming increasingly more prevalent in the developing world. The myocardial injuries caused by AMI usually cause the patient to have severe arrhythmias that, in many cases, culminate in the patient’s death. Therefore, researchers look for strategies that can mitigate both myocardial injuries and cardiac arrhythmias^1,2^.

The cardiac muscle can tolerate short periods of severe and total ischemia, such as those caused by coronary vasospasms, without myocyte cell death^1^. However, the greater the duration and/or severity of cardiac ischemia, the more extensive will be the myocardial damage, as well as susceptibility to further injury during reperfusion^1^. The combined damage caused by the obstruction and subsequent coronary clearing is known as a cardiac ischemia-reperfusion (CIR) injury. It is well known that these lesions compromise cardiac structure and function and are responsible for many clinical sequelae typical of AMI^1^.

It is well established that the precise adjustment of cytosolic ([Ca^2^]c) and mitochondrial ([Ca^2^]m) concentrations of Ca^2^ in cardiomyocytes is crucial to maintain the strength and frequency of cardiac contractions. Then, dysregulation of Ca^2^ homeostasis in cardiomyocytes caused by CIR decisively contribute to cardiac dysfunction in AMI^2-5^. This ionic imbalance produced by CIR in cardiomyocytes, especially the unbalancing of [Ca^2^]c and [Ca^2^]m has unsurprisingly been implicated as a major cause of severe and lethal cardiac arrhythmias due to ischemic heart disease^2^.

In healthy cardiomyocytes, cytosolic Ca^2^ overload is prevented by Ca^2^-ATPase SERCA2a-mediated Ca^2^ uptake from the cytosol into the sarcoplasmic reticulum (SER)^3^. Meanwhile, in CIR-injured cardiomyocytes, increased generation of free radicals compromises the selective permeability of the inner mitochondrial membrane, leading to a drastic Ca^2^ influx mediated mainly by mitochondrial Ca^2^ uniporter (MCU) that collapses the mitochondrial function, severely reducing ATP production^4^. In addition, the reduction in SERCA2a activity, as well as its expression, further exacerbates the cytosolic and mitochondrial Ca^2^ overload in cardiomyocytes^5^. Ionic imbalance produced during ischemia produces a considerable increment in [Ca^2^]c in cardiomyocytes, which generate a significant increase in Ca^2^ uptake by mitochondria, thus aggravating mitochondrial Ca^2^ overload in a positive feedback loop^6,7^. This Ca^2^ overload that collapses the mitochondrial function dramatically compromises the excitation-contraction coupling, favoring the development of fatal cardiac arrhythmias^8^.

Due to the crucial role of ionic imbalance induced by CIR in cardiomyocytes, particularly in [Ca^2^]m, in the development of most severe adverse effects, pharmacological strategies with the cardioprotective potential to attenuate these changes are highly desirable. Therefore, in this study we evaluated whether the attenuation of mitochondrial Ca^2^ overload produced by pharmacological blockade of MCU with Ruthenium Red (RuR) protects the myocardium against injuries caused by CIR.

## Methods

All experimental protocols used in this study were approved by the Ethics Committee of the Universidade Federal de São Paulo (#1130/11 and #0065/12).

Seventy-three male Wistar rats (320 ± 30 g) were kept at 21 ± 2°C with a 12:12-h light/dark cycle and had *ad libitum* access to water and food during the experimental procedures. Rats were randomly divided into four groups:

- **CIR group:** 11 rats underwent surgery to induce cardiac ischemia (10 min), with subsequent removal of the wire (cardiac reperfusion) and electrocardiogram (ECG) monitoring for 120 min;
- **CIR+SS group:** 22 rats were sham-treated with 0.9% saline solution (SS), administrated intravenously immediately before (n = 11) or after (n = 11) induction of cardiac ischemia, with subsequent cardiac reperfusion and ECG monitoring for 120 min. No differences were observed between sham-treatment before or after CIR;
- **RuR+CIR group:** 16 rats were treated with intravenous RuR (1 mg/kg, n = 8; or 3 mg/kg, n = 8), immediately before induction of cardiac ischemia (10 min), with subsequent cardiac reperfusion and ECG monitoring for 120 min;
- **CIR+RuR group:** 18 rats underwent surgery to induce cardiac ischemia (10 min) and were subsequently treated with intravenous RuR (1 mg/kg, n = 10; 3 mg/kg, n = 8), before 120 min of ECG-monitored reperfusion.

### Surgical protocol for induction of CIR

CIR protocol was performed as previously described^9^. After anesthesia with urethane (1.25 g/kg) and under mechanical ventilation, the rats were submitted to left-thoracotomy. A surgical tourniquet was tied around the left anterior descending coronary artery. After 10 min of coronary occlusion, reperfusion was obtained by cutting the suture. Successful surgical obstruction of the coronary artery was confirmed through observation of electrocardiogram (ECG) changes^14,15^.

### ECG recordings and analysis

ECG was monitored and recorded from the beginning of the stabilization period with a commercial acquisition system (AqDados 7.02; Lynx Tecnologia Ltda., Brazil) as reported earlier^9^. To assess heart rate, incidences of reperfusion-induced ventricular arrythmia (VA), atrioventricular block (AVB) and lethality (LET), raw data were analyzed with a commercial software package included in the acquisition system (AqDAnalysis 7, Lynx Tecnologia Ltda.). Ventricular fibrillation, Torsades de pointes and ventricular tachycardia were grouped as VA. ECG measurements were analyzed as stated by the Lambeth conventions^16,17^.

### Data analysis

Incidences of VA, AVB and LET in rats submitted to CIR are reported as percentages and were compared using Fisher’s exact test. Values were considered significant when p ˂ 0.05. Statistical analyses were performed with the Prism 5.0 software (GraphPad, USA).

## Results

**Figure 1:**
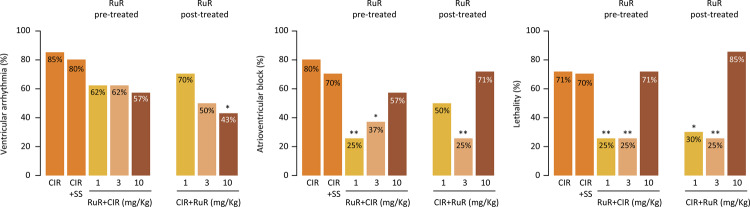
Effects of MCU blocker ruthenium red (RuR) on incidences of ventricular arrhythmia (VA), atrioventricular block (AVB) and lethality (LET) in rats from CIR, CIR + SS, RuR + CIR and CIR + RuR groups. In CIR + SS, rats were treated with 0.9% saline solution (SS) administrated intravenously immediately before ischemia. In RuR + CIR and CIR + RuR groups, rats were treated with RuR (1, 3 and 10 mg/kg) administrated intravenously immediately before (pre-treated) and after (post-treated) ischemia. Statistically different from the CIR group according to Fisher’s exact test (* p < 0.05; ** p < 0.005).

In the CIR group, incidences of VA, AVB and LET were 85%, 80% and 70%, respectively. No significant differences were observed between CIR and CIR+SS group (80%, 70% and 70%, respectively). Meanwhile, treatment of rats with either 1 mg/kg or 3 mg/kg RuR before CIR did not affect VA incidence, but effectively reduced the incidence of AVB and LET. Incidences of VA, AVB and LET in rats treated with 1 mg/kg RuR were, respectively, 62% (p > 0.05), 25% (p < 0.005) and 25% (p < 0.05). No further reductions were observed in VA, AVB and LET incidences in rats treated with 3 mg/kg RuR, with respective incidence of 62% (p > 0.05), 37% (p < 0.05) and 25% (p < 0.05). On the other hand, a higher RuR dose of 10 mg/kg had no cardioprotective effect on VA, AVB and LET, with incidences of 57%, 57% and 71%, respectively – no different than those of CIR-only animals (p > 0.05) (Fig. 1; RuR pre-treated column).

We also investigated whether RuR also had a cardioprotective effect when administered immediately after the CIR protocol. We observed similar, albeit somewhat less marked cardioprotection with treatment of rats with 1 mg/kg RuR after CIR: no effect on VA or AVB incidence was observed, but LET was significantly reduced. Incidences of VA, AVB and LET for 1 mg/kg RuR were, respectively, 70% (p > 0.05), 50% (p > 0.05) and 30% (p < 0.05) (Fig. 1). Dose of 3 mg/kg RuR again did not affect VA incidence, but reduced AVB and LET when compared to controls, with respective incidences of 50% (p > 0.05), 25% (p < 0.005) and 25% (p < 0.005). Finally, the higher dose of 10 mg/kg RuR decreased VA incidence to 43% (p < 0.05), but was mostly ineffective in producing cardioprotective effects, with incidences of AVB and LET of 71% and 85%, no different than those obtained in CIR group (p > 0.05) (Fig. 1; RuR post-treated column).

## Discussion

In this study, we showed that the incidences of VA, AVB and LET induced by CIR were significantly reduced by MCU blocker RuR treatment, suggesting that the attenuation of mitochondrial Ca^2^ overload due to pharmacological blockade of MCU can protect the myocardium against injuries caused by CIR. This pharmacological strategy could be important to attenuate myocardial injuries and cardiac arrhythmias in patients with AMI.

Several evidences suggest that inhibition of mitochondrial Ca^2^ influx mediated by MCU in CIR could attenuate ionic imbalances in cardiomyocytes by reducing Ca^2^ overload in the mitochondrial matrix, thus reducing the incidence of cardiac arrhythmias and lethality^18^. For example, García-Rivas *et al*.^22^ showed that selective MCU blocker Ru360 (15-50 nmol/kg, i.v.), a RuR analog, given 30 min before ischemia (5 min) followed by reperfusion (5 min), significantly reduced the incidence of arrhythmias and the hemodynamic dysfunctions caused by cardiac CIR in rats. Zhang *et al*.^23^ showed that isolated hearts of rats submitted to a hypoxia (30 min) and reoxygenation (120 min) protocol showed significant reduction in contractile activity, accompanied by an increase in the release of lactate dehydrogenase, both of which were prevented by RuR (5 μM) and exacerbated by MCU activator spermine (20 μM) administered 5 min prior to hypoxia. In this study, we showed that RuR (1 and 3 mg/kg, i.v.) was able to reduce the incidence of AVB and LET in animals with a more severe CIR-induced lesion *in vivo* (10 min ischemia and 120 min reperfusion), even when it was administered after CIR, which might be relevant in a clinical setting were pre-treatment was not viable.

The ionic imbalance in CIR-affected cardiomyocytes significantly affects MCU-mediated influx of Ca^2^, generating an bioenergetic collapse in cardiomyocytes due to Ca^2^ overload in the mitochondria matrix^24^. The main possible mechanism for the cardioprotective effects observed on CIR-induced arrhythmias and LET with RuR in this study is its specific inhibition of MCU, decreasing influx of Ca^2^ into the mitochondrial matrix and Ca^2^ overload, preserving ATP production and functional integrity of the mitochondria, likely mechanisms for cardiac arrhythmias caused by CIR^27^. Additionally, RuR can also affect [Ca^2^]m indirectly, reducing [Ca^2^]c through inhibition of Ca^2^ release from the SER due to interaction of with ryanodine receptors (RyR), tubulin and Ca^2^-ATPases^20,28,29^.

Our results also showed that administration of high doses of RuR (10 mg/kg) have no cardioprotective effect compared to the CIR group and, on the contrary, tended to increase lethality, indicating that doses above 10 mg/kg may produce cardiotoxic effects. Indeed, in isolated rat hearts or cardiomyocytes, administration of 5 μM RuR reduced [Ca^2^]c due to inhibition of Ca^2^ release from SER mediated by RyR, but higher concentrations (10 to 50 μM) produced spontaneous contractions and contracture, through mechanisms that remain unclear, but may include mitochondrial dysfunction independent of RuR’s effect on MCU.

Attenuating [Ca^2^]m overload by MCU blockade could play an important role in cardioprotection against CIR damage by attenuating ionic imbalance and mitochondrial bioenergetic collapse in cardiomyocytes, thus preventing cardiac contractile function failure and fatal arrhythmias^30^. Therefore, drugs capable of blocking MCU may constitute an effective cardioprotective strategy in the aftermath of AMI.

## Conclusion

The significant reduction of the incidence of CIR-induced VA, AVB and LET produced by the treatment with RuR indicates that the attenuation of mitochondrial Ca^2^ overload produced by pharmacological blockade of MCU can protect the myocardium against injuries caused by CIR. This finding could open a new avenue in AMI treatment.

## Footnotes

1 Research performed at Laboratory of Autonomic and Cardiovascular Pharmacology, Department of Pharmacology, Universidade Federal de São Paulo (UNIFESP), Brazil. Part of PhD degree thesis, Postgraduate Program in Pharmacology, UNIFESP. Tutor: Prof. Dr. Afonso Caricati-Neto.

## References

[1] Verma S, Fedak PWM, Weisel RD, Butany J, Rao V, Maitland A, Li RK, Dhillon B, Yau TM (2002). Fundamentals of reperfusion injury for the clinical cardiologist. Circulation.

[2] Yang K-C, Bonini MG, Dudley SC (2014). Mitochondria and arrhythmias. Free Radic Biol Med.

[3] Akolkar G, Pande J, Samson SE, Grover AK (2012). Thapsigargin decreases the Na+- Ca2+ exchanger mediated Ca2+ entry in pig coronary artery smooth muscle. Biochim Biophys Acta Biomembr.

[4] O’Rourke B, Cortassa S, Aon MA (2005). Mitochondrial ion channels: gatekeepers of life and death. Physiology.

[5] Talukder MAH, Yang F, Nishijima Y, Chen C, Kalyanasundaram A, Periasamy M, Zweier JL (2009). Reduced SERCA2a converts sub-lethal myocardial injury to infarction and affects postischemic functional recovery. J Mol Cell Cardiol.

[6] Halestrap AP, Clarke SJ, Khaliulin I (2007). The role of mitochondria in protection of the heart by preconditioning. Biochim Biophys Acta.

[7] Williams GSB, Boyman L, Chikando AC, Khairallah RJ, Lederer WJ (2013). Mitochondrial calcium uptake. Proc Natl Acad Sci U S A.

[8] Xie L-HH, Weiss JN (2009). Arrhythmogenic consequences of intracellular calcium waves. Am J Physiol Heart Circ Physiol.

[9] Menezes-Rodrigues FS, Errante PR, Ferreira RM, Tavares JGP, Paula L, Araújo EA, Govato TCP, Tikazawa EH, Reis MDCM, Luna-Filho B, Ferraz RRN, Oliveira-Júnior IS, Taha MO, Caricati-Neto A (2018). Cardioprotective effect of lipstatin derivative orlistat on normotensive rats submitted to cardiac ischemia and reperfusion. Acta Cir Bras.

[10] Menezes-Rodrigues FS, Errante PR, Tavares JGP, Ferraz RRN, Gomes WJ, Taha MO, Scorza CA, Scorza FA, Caricati-Neto A (2019). Pharmacological modulation of b-adrenoceptors as a new cardioprotective strategy for therapy of myocardial dysfunction induced by ischemia and reperfusion. Acta Cir Bras.

[11] Tavares JG, Vasques ER, Arida RM, Cavalheiro EA, Cabral FR, Torres LB, Menezes-Rodrigues FS, Jurkiewicz A, Caricati-Neto A, Godoy CM, Gomes da Silva S (2015). Epilepsy-induced electrocardiographic alterations following cardiac ischemia and reperfusion in rats. Braz J Med Biol Res.

[12] Tavares JGP, Menezes-Rodrigues FS, Vasques ER, Maia-Reis MC, Paula L, Luna-Filho B, Errante PR, Caricati-Neto A, Bergantin LB (2017). A simple and efficient methodology for the study of cardioprotective drugs in animal model of cardiac ischemia-reperfusion. J Mol Imaging Dyn.

[13] Tavares JGP, Errante PR, Govato TCP, Vasques ER, Ferraz RRN, Taha MO, Menezes-Rodrigues FS, Caricati-Neto A (2018). Cardioprotective effect of preconditioning is more efficient than postconditioning in rats submitted to cardiac ischemia and reperfusion. Acta Cir Bras.

[14] Di Diego JM, Antzelevitch C (2003). Cellular basis for ST-segment changes observed during ischemia. J Electrocardiol.

[15] Yan G-X, Lankipalli RS, Burke JF, Musco S, Kowey PR (2003). Ventricular repolarization components on the electrocardiogram: cellular basis and clinical significance. J Am Coll Cardiol.

[16] Walker MJ, Curtis MJ, Hearse DJ, Campbell RW, Janse MJ, Yellon DM, Cobbe SM, Coker SJ, Harness JB, Harron DW (1988). The Lambeth Conventions: guidelines for the study of arrhythmias in ischaemia infarction, and reperfusion. Cardiovasc Res.

[17] Huggins CE, Bell JR, Pepe S, Delbridge LMD (2008). Benchmarking ventricular arrhythmias in the mouse--revisiting the “Lambeth Conventions” 20 years on. Heart Lung Circ.

[18] Dedkova EN, Blatter LA (2008). Mitochondrial Ca2+ and the heart. Cell Calcium.

[19] Di Lisa F, Schulz R, Murphy E (2011). Preface to mitochondria and cardioprotection. Biochim Biophys Acta.

[20] Griffiths EJ (2009). Mitochondrial calcium transport in the heart: physiological and pathological roles. J Mol Cell Cardiol.

[21] Perrelli M-G, Pagliaro P, Penna C (2011). Ischemia/reperfusion injury and cardioprotective mechanisms: role of mitochondria and reactive oxygen species. World J Cardiol.

[22] García-Rivas G de J, Carvajal K, Correa F, Zazueta C (2006). Ru360, a specific mitochondrial calcium uptake inhibitor, improves cardiac post-ischaemic functional recovery in rats in vivo. Br J Pharmacol.

[23] Zhang S, Gao Q, Cao C, Bruce IC, Xia Q (2006). Involvement of the mitochondrial calcium uniporter in cardioprotection by ischemic preconditioning. Life Sci.

[24] Duchen MR (2004). Mitochondria in health and disease: perspectives on a new mitochondrial biology. Mol Aspects Med.

[25] Duchen MR, Verkhratsky A, Muallem S (2008). Mitochondria and calcium in health and disease. Cell Calcium.

[26] Duchen MR, Szabadkai G (2010). Roles of mitochondria in human disease. Essays Biochem.

[27] Brown DA, O’Rourke B (2010). Cardiac mitochondria and arrhythmias. Cardiovasc Res.

[28] Hajnóczky G, Csordás G, Das S, Garcia-Perez C, Saotome M, Sinha Roy S, Yi M (2006). Mitochondrial calcium signalling and cell death: approaches for assessing the role of mitochondrial Ca2+ uptake in apoptosis. Cell Calcium.

[29] Zucchi R, Ronca-Testoni S (1997). The sarcoplasmic reticulum Ca2+ channel/ryanodine receptor: modulation by endogenous effectors, drugs and disease states. Pharmacol Rev.

[30] Cao C, Yan WY, Liu J, Kam KW, Zhan SZ, Sham JS, Wong TM (2006). Attenuation of mitochondrial, but not cytosolic, Ca2+ overload reduces myocardial injury induced by ischemia and reperfusion. Acta Pharmacol Sin.

